# Genomic Characterization of Carbapenem-Resistant *Acinetobacter baumannii* (OXA-23) and *Klebsiella pneumoniae* (KPC-2) Causing Hospital-Acquired Infections in Dogs

**DOI:** 10.3390/antibiotics14060584

**Published:** 2025-06-06

**Authors:** Isabela Pádua Zanon, João Victor Ferreira Campos, Yasmin Gonçalves de Castro, Isadora Maria Soares de Melo, Flávia Figueira Aburjaile, Bertram Brenig, Vasco Azevedo, Rodrigo Otávio Silveira Silva

**Affiliations:** 1 Departamento de Medicina Veterinária Preventiva, Universidade Federal de Minas Gerais (UFMG), Belo Horizonte 30720440, Brazil; 2 Institute of Veterinary Medicine, University of Göttingen, 37077 Göttingen, Germany; 3 Instituto de Ciências Biológicas (ICB), Universidade Federal de Minas Gerais (UFMG), Belo Horizonte 30720440, Brazil

**Keywords:** healthcare-associated infection, companion animals, carbapenem-resistance, ESKAPE, KPC-2, OXA-23, *Acinetobacter baumannii*, *Klebsiella pneumoniae*

## Abstract

**Background/Objectives:** Antimicrobial resistance is a major global health threat. Among the most problematic pathogens are carbapenem-resistant *Acinetobacter baumannii* and *Klebsiella pneumoniae*, which are significant causes of mortality in humans, particularly in the context of nosocomial infections. In companion animals, these bacteria have been reported mainly as colonizers of healthy animals or, less frequently, in community-acquired infections. However, no confirmed cases of healthcare-associated infections caused by these species have been documented in this population. This study reports the first confirmed fatal cases of infection with carbapenem-resistant *A. baumannii* and KPC-producing *K. pneumoniae* in dogs. **Methods**: Three hospitalized dogs developed infections associated with distinct anatomical devices, including a venous catheter, an endotracheal tube, and a Penrose drain. Bacterial isolation followed by antimicrobial susceptibility testing identified carbapenem-resistant *A. baumannii* and *K. pneumoniae*. The isolates were subsequently subjected to additional antimicrobial resistance tests and whole-genome sequencing (WGS). **Results**: WGS confirmed the presence of the OXA-23 carbapenemase gene in both *A. baumannii* isolates and the KPC-2 carbapenemase gene was detected in the *K. pneumoniae* strain. All three strains exhibited resistance to multiple antimicrobial classes, including β-lactams (amoxicillin-clavulanic acid, ampicillin, cephalotin, piperacillin-tazobactam, cefoxitin, ceftiofur, cefotaxime, ertapenem, imipenem and meropenem), aminoglycosides (gentamicin, neomycin), tetracyclines (doxycycline, tetracycline and oxytetracycline), fluoroquinolones (ciprofloxacin, enrofloxacin), and folate pathway antagonists (trimethoprim-sulfamethoxazole). Multilocus sequence typing identified two high-risk clones: *K. pneumoniae* ST340 (CC258) and *A. baumannii* ST15 (CC15). Single nucleotide polymorphism analysis confirmed a high degree of genetic similarity between these isolates and strains previously associated with human infections in Brazil. **Conclusions**: These findings provide the first evidence of fatal, healthcare-associated infections caused by these multidrug-resistant pathogens in dogs and underscore the need to strengthen surveillance and infection control practices in veterinary hospitals. Furthermore, the results raise concerns about the potential of companion animals to act as reservoirs for multidrug-resistant organisms of public health relevance.

## 1. Introduction

*Acinetobacter baumannii* and *Klebsiella pneumoniae* are opportunistic Gram-negative bacilli recognized as major etiological agents of healthcare-associated infections, particularly in immunocompromised and critically ill patients admitted to intensive care units [1,2,3]. In this context, the emergence of multidrug-resistant strains, particularly those resistant to carbapenems, poses a major clinical challenge, as it significantly limits therapeutic options and complicates clinical management [4,5]. Carbapenems are recognized as last-resort antimicrobials, primarily reserved for the treatment of infections caused by multidrug-resistant pathogens [6,7,8]. Moreover, the World Organization for Animal Health (WOAH) emphasizes that the use of this antimicrobial class should be restricted exclusively to human medicine in order to safeguard its efficacy [9]. Therefore, the detection of strains resistant to this class of antimicrobials in veterinary hospital settings is a cause for concern, as it underscores the dissemination of resistant isolates that pose a potential threat to public health [10].

Current studies have reported the presence of these pathogens in companion animals and veterinary environments, including mostly *A. baumannii* carrying *bla*_OXA-23_ and *bla*_OXA-51_ and Enterobacteriaceae KPC-producing isolates [11,12,13,14,15,16,17,18,19,20]. However, none have addressed the occurrence of carbapenem-resistant strains causing nosocomial infections in companion animals. Moreover, there is no available information regarding two clinically significant sequence types—*K. pneumoniae* ST340 and *A. baumannii* ST15—which are recognized as relevant epidemic clones circulating in several countries, including in Brazil [21,22,23,24]. Thus, the aim of this study was to describe three fatal cases of infection caused by carbapenemase-producing *A. baumannii* and *K. pneumoniae* in dogs, as well as to identify their antimicrobial resistance determinants and investigate their phylogenetic relationships with strains previously isolated from infected humans.

## 2. Results

### 2.1. Bacterial Isolation and Antimicrobial Susceptibility

In all three cases, the samples collected resulted in the growth of pure colonies on MacConkey agar and Blood agar after 24 h of incubation. Species identification confirmed *K. pneumoniae* in Case 1 (B145) and *A. baumannii* in Cases 2 and 3 (B146 and BR250, respectively). In Case 1, the *K. pneumoniae* isolate exhibited resistance to penicillins (amoxicillin-clavulanic acid and ampicillin), third-generation cephalosporins (ceftiofur and cefotaxime), fluoroquinolones (ciprofloxacin and enrofloxacin), tetracyclines (doxycycline and oxytetracycline), aminoglycosides (gentamicin and neomycin), nitrofurans (nitrofurantoin), and carbapenems (ertapenem). It remained susceptible only to phenicols (florfenicol) and folate pathway antagonists (trimethoprim-sulfamethoxazole). Additionally, the isolate showed a minimum inhibitory concentration (MIC) of 12 µg/mL for ertapenem and 0.125 µg/mL for colistin, being categorized as resistant and intermediate, respectively.

*A. baumannii* isolate B146 (Case 2) exhibited resistance to penicillins (piperacillin-tazobactam), fluoroquinolones (ciprofloxacin), carbapenems (imipenem and meropenem), aminoglycosides (gentamicin), folate pathway antagonists (trimethoprim-sulfamethoxazole), and tetracyclines (tetracycline) in the disk diffusion method, displaying susceptibility only to third-generation cephalosporins (ceftazidime). This isolate presented a MIC of 4 µg/mL for meropenem and 0.125 µg/mL for colistin, both falling within the intermediate range. Conversely, isolate BR250 (Case 3) was resistant to penicillins (piperacillin-tazobactam), third-generation cephalosporins (ceftazidime), fluoroquinolones (ciprofloxacin), carbapenems (imipenem), folate pathway antagonists (trimethoprim-sulfamethoxazole), and tetracyclines (tetracycline). It remained susceptible only to aminoglycosides (gentamicin). The isolate showed a MIC of >32 µg/mL for meropenem and 0.047 µg/mL for colistin, being classified as resistant and intermediate, respectively. The three isolates obtained in this study were all classified as multidrug resistant [5].

### 2.2. Comparative Genomics

#### 2.2.1. Presence and Type of Carbapenemase Genes

**Figure 1:**
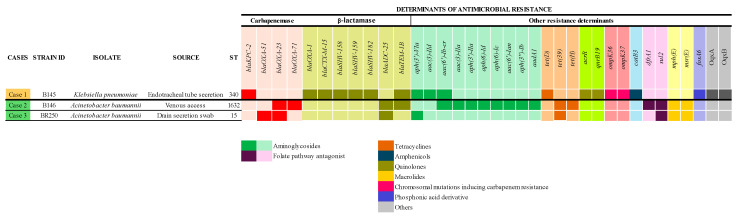
Multilocus sequence typing (MLST) and antimicrobial resistance determinant genes detected in clinical isolates of *K. pneumoniae* and *A. baumannii*.

The *K. pneumoniae* isolate harbored the *bla*_KPC-2_ gene, which encodes a class A carbapenemase commonly found in Enterobacteriaceae [6]. Additionally, this isolate exhibited point mutations in the genes encoding the OmpK36 and OmpK37 porins (*ompK36* and *ompK37*). These mutations likely reduce the permeability of the outer membrane to carbapenem antibiotics, thereby contributing to resistance to these compounds. Both *A. baumannii* isolates harbored the *bla*_OXA-23_ gene, which encodes a class D carbapenem-hydrolyzing β-lactamase (Figure 1). Additionally, the *A. baumannii* BR250 also carried the *bla*_OXA-51_ gene, another relevant carbapenemase.

#### 2.2.2. Other Determinants of Antimicrobial Resistance

*K. pneumoniae* isolate B145 (Case 1) carried resistance genes for multiple antimicrobial classes (Figure 1). Notably, it harbored several *bla* and SHV genes encoding β-lactamases, which confer resistance to non-carbapenem β-lactams, including SHV-158 and SHV-159 (Figure 1), which are Extended-Spectrum Beta-Lactamases (ESBL) [25,26]. Additionally, the isolate possessed *oqxA* and *oqxB*, encoding a multidrug efflux pump that contributes to resistance against quinolones, folate pathway antagonists, and phenicols. Furthermore, multiple determinants of fluoroquinolone resistance were identified, including point mutations in the *acrR* gene, the presence of plasmid-mediated quinolone resistance (PMQR) gene (*qnrB19)*, and the *aac(6′)-Ib-cr* gene, which encodes an aminoglycoside acetyltransferase that also reduces fluoroquinolone susceptibility in clinical bacterial isolates [27].

Both *A. baumannii* isolates carried resistance determinants associated with tetracyclines (*tet(39)*), folate pathway antagonists (*sul2*), and macrolides (*msr(E)* and *mph(E)*). In isolate B146, a particularly high number of aminoglycoside resistance genes was also observed (Figure 1).

#### 2.2.3. Mobile Genetic Elements

Six plasmid replicons were detected in the sequenced *K. pneumoniae* strain, and two of them carried antimicrobial resistance determinants (Supplementary File S2). Additionally, this isolate also harbored the insertion sequence (IS) ISCfr1, which carries the *aac(3)-IId* gene associated with aminoglycoside resistance. In the two *A. baumannii* isolates, no plasmids were identified. On the other hand, relevant ISs were detected, including ISVsa3, which harbors the *sul2* gene associated with resistance to folate pathway antagonists (Supplementary File S2).

#### 2.2.4. MLST, KL-Typing, OCL-Typing and SNP Analysis

**Figure 2:**
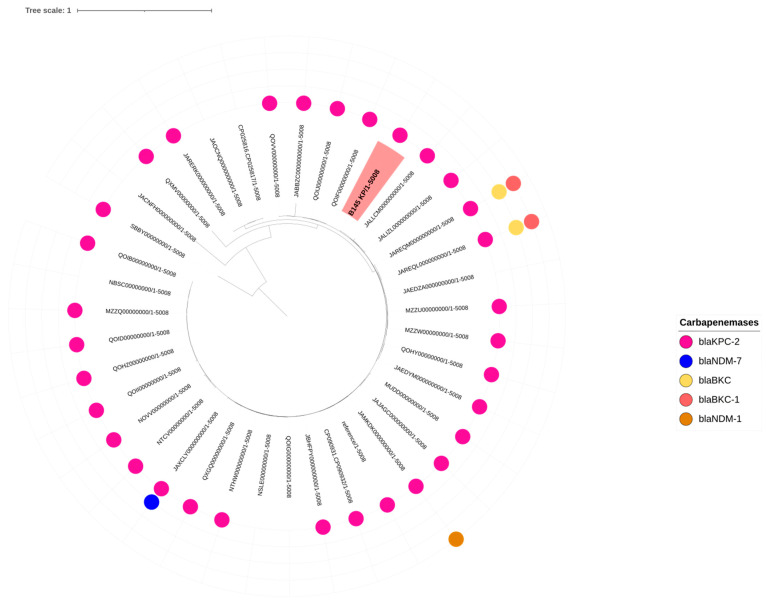
Single nucleotide polymorphism (SNP) analysis showing the genetic relationship between the B145 *K. pneumoniae* isolate and human isolates belonging to ST340 from Brazil. The phylogenetic tree was generated online using iTOL [28] with midpoint rooting.

MLST analysis revealed that the *K. pneumoniae* isolate belonged to sequence type (ST) 340, which is part of clonal complex (CC) 258. Meanwhile, the *A. baumannii* isolates B146 and BR250 were assigned to ST15 (CC15*^Pas^*/CC103^Ox^) and ST1632, respectively. The K and O loci of the *K. pneumoniae* isolate were identified as KL15 and O4, respectively. The SNP analysis of the *K. pneumoniae* isolate revealed high similarity to strains previously isolated from infected humans in Brazil, with differences ranging from 59 up to 2228 SNPs (Supplementary File S3) (Figure 2).

**Figure 3:**
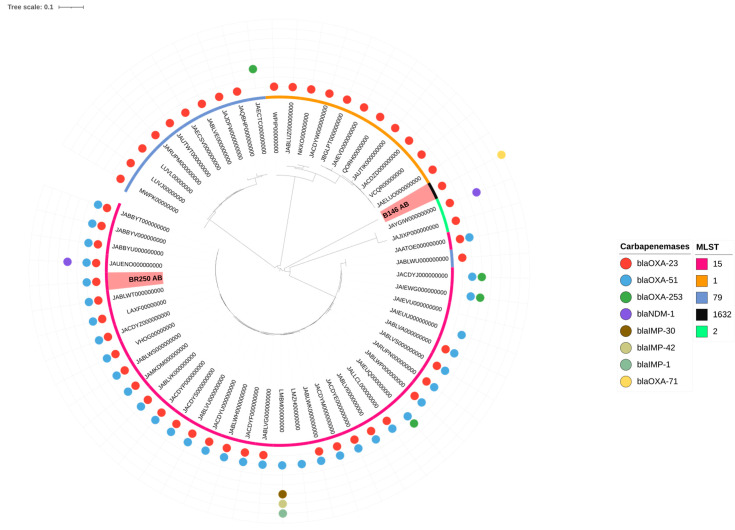
Single nucleotide polymorphism (SNP) analysis illustrating the genetic relationships between *A. baumannii* isolates B146 (Case 2) and BR250 (Case 3) and human ST15, ST79, ST1, and ST2 strains from Brazil. The phylogenetic tree was generated online using iTOL [28] with midpoint rooting.

Additionally, SNP analysis of the *A. baumannii* isolate BR250 (Case 3) revealed high genetic similarity to strains previously isolated from infected humans in Brazil, with differences ranging from 13 to 968 SNPs among strains belonging to ST15. In contrast, SNP analysis of the B146 *A. baumannii* (Case 2) isolate showed greater diversity, with differences ranging from at least 14,023 SNPs (Supplementary File S3) (Figure 3).

## 3. Discussion

Carbapenem-resistant *A. baumannii* and *K. pneumoniae* are globally distributed and are associated with human infections, particularly in intensive care units [1,29]. According to the World Health Organization (WHO), carbapenem-resistant Enterobacterales are listed in the critical priority category due to the urgent need for the development of new antibiotics [30]. The multidrug-resistant profile of these isolates limits treatment options and can lead to fatal outcomes [31,32]. Data on antimicrobial resistance and phylogenetic relationships between *A. baumannii* and *K. pneumoniae* isolates from animals are scarce and most are limited to studies of colonized animals or veterinary environments [11,17,33,34]. Furthermore, to the best of our knowledge, no cases have yet been reported of infections caused by *A. baumannii* ST15 and *K. pneumoniae* ST340 in companion animals—two globally disseminated STs responsible for human infections [21,22,23,24,35,36].

In this study, we describe three cases—two of them fatal—caused by carbapenem-resistant *A. baumannii* and *K. pneumoniae* in dogs hospitalized at a veterinary hospital. Both *A. baumannii* isolates harbored the acquired carbapenemase gene *bla*_OXA-23_, which encodes a class D β-lactamase (oxacillinase) commonly associated with carbapenem resistance and widely reported in human clinical isolates across multiple geographic regions [37]. In addition, the *A. baumannii* isolates carried the insertion sequence ISAba1, which is known to enhance the expression of *bla*_OXA-23_ and the intrinsic gene *bla*_OXA-51_ (another relevant carbapenemase) by providing a strong promoter upstream of both genes [38,39]. Conversely, the *K. pneumoniae* isolate harbored the *bla*_KPC-2_ gene, which is globally disseminated and associated with high-risk multidrug-resistant clones [40,41,42,43]. In addition, this isolate also carried the *bla*_CTX-M-15_ gene and SHV-type β-lactamase genes, such as *bla*_SHV-158_ and *bla*_SHV-159_. As previously mentioned, carbapenems are considered the first-line therapy for infections caused by ESBL-producing Enterobacteriaceae [44,45]. In this context, the detection of such findings in veterinary clinical settings is particularly alarming, as the presence of carbapenemase-producing organisms significantly compromises therapeutic efficacy, often leading to prolonged treatment courses and an increased risk of therapeutic failure [6,46,47].

Two clinically significant sequence types (STs) were identified in the *K. pneumoniae* and *A. baumannii* isolates, ST340 and ST15, respectively. *K. pneumoniae* ST340 belongs to clonal complex 258 (CC258), one of the most globally disseminated lineages, highly adapted to human populations and frequently associated with nosocomial infections [48,49]. Moreover, ST340 has previously been reported as the causative agent of hospital outbreaks in Brazil [24,50,51]. Interestingly, previous works documented the presence of *K. pneumoniae* from CC258 in animals [18,52,53], but the present report seems to be the first report of *K. pneumoniae* ST340 in animals.

One of the *A. baumannii* isolates (from Case 3) was assigned to ST15 (CC15), a clonal complex that, although less prevalent than the epidemic clonal complexes (CC1 and CC2), is frequently reported in South America, including Brazil [21,22,35,54,55,56,57,58,59,60]. These findings highlight the spread of globally significant high-risk clones among companion animals, underscoring their potential role in the transmission of antimicrobial-resistant strains to humans. In contrast, the *A. baumannii* B146 (Case 2) isolate was assigned to ST1632, a poorly characterized sequence type that has not yet been reported as a cause of infection in either humans or animals.

SNP analysis revealed a high genetic similarity between the *K. pneumoniae* isolate and clinical strains recovered from infected human patients, with a minimum difference of only 59 SNPs. This finding suggests that strains from this clonal complex—already known to be endemic in the human population in Brazil and previously detected in veterinary hospital environments [23,61]—can be shared with and also infect companion animals. A study conducted in Portugal has already provided evidence of strain sharing of *K. pneumoniae* between healthy humans and their cohabiting companion animals [62]. In a hospital environment, this scenario becomes even more concerning, as it often involves multidrug-resistant and hypervirulent isolates, such as the one described in the present study. Similarly, SNP analysis of the *A. baumannii* isolate belonging to ST15 revealed high genomic similarity to strains of the same ST previously isolated from infected humans in Brazil, with a minimum difference of only 13 SNPs. This sequence type, already recognized as endemic among *A. baumannii* isolates in Latin America, including Brazil [21,63,64], poses a public health concern due to its detection in a veterinary hospital and its close genetic relatedness to strains previously isolated from infected humans.

Antimicrobial resistance is already a reality in human medicine, representing a global concern due to its economic and social impact [65,66,67]. The detection of resistant strains is closely associated with worse patient outcomes and, consequently, higher mortality rates [68,69,70]. Since 2017, WHO has categorized various bacterial pathogens into different priority levels [71]. In veterinary medicine, the occurrence of these pathogens, including multidrug-resistant strains, is increasingly observed and warrants attention [72]. Interestingly, food animals are well-recognized reservoirs of antimicrobial-resistant microorganisms, and consequently, most public policies and research efforts have traditionally focused on these species [73,74]. However, the present study highlights the need for increased attention to companion animals. Although cats and dogs are generally less likely to receive antimicrobials compared to food animals, they are occasionally treated with critically important drugs, such as vancomycin and carbapenems. Additionally, they may be hospitalized, including in intensive care units, conditions that substantially increase the risk of colonization or infection with multidrug-resistant organisms such as KPC-producing bacteria and carbapenem-resistant *A. baumannii*. Anyway, for both companion and food animals, it is clear that antimicrobial resistance in animals must be addressed within the One Health approach, as epidemiology in animals is closely linked to that in humans [75,76].

Beyond surveillance approaches, reassessing clinical practices is imperative, including the stepwise use of antimicrobials for each clinical case, in accordance with established international guidelines [77,78,79,80,81]. Considering these concerns, a Hospital Infection Control Committee was established at the veterinary facility where these cases occurred, with the primary responsibility of monitoring all cases of nosocomial infections caused by multidrug-resistant pathogens, particularly those resistant to carbapenems.

This study has some limitations. Although it highlights the occurrence of nosocomial infections caused by carbapenem-resistant strains and raises awareness of this emerging threat, it is based on only three cases. Therefore, broader investigations are needed to better assess the frequency of such infections and to identify potential risk factors. In addition, necropsies were not performed because the owners did not authorize the procedures. This limited our ability to fully characterize the pathological findings, which would have strengthened the clinical interpretation of the cases.

## 4. Materials and Methods

### 4.1. Case 1

An eleven-year-old neutered male Lhasa Apso dog (B145), positive for leishmaniasis and living with five other dogs, was presented to the veterinary hospital with suspected chronic pancreatitis. The patient was immediately admitted to the hospital, where he received antimicrobial therapy with clindamycin, doxycycline, and ceftriaxone. Vasoactive therapy and exogenous oxygen administration were also initiated. Two days after admission, purulent secretion was observed in the endotracheal tube. The animal showed signs of sepsis and died three days after admission. A swab of endotracheal tube secretion was aseptically collected for bacteriological analysis. Informed consent was obtained from the owner for this case report but not for a necropsy.

### 4.2. Case 2

A thirteen-year-old intact male Golden Retriever (B146), diagnosed with chronic kidney disease and hip dysplasia, was admitted to the veterinary hospital with a history of lethargy, polyuria, dyschezia and scrotal sac lesion. On clinical examination, facial edema and submandibular abscess were noted. Ultrasonographic examination revealed lymphadenomegaly and acute hepatitis, as well as testicular asymmetry due to a possible neoplastic process in the left testicle. The patient was hospitalized in the intensive care unit and antimicrobial therapy was initiated. Initial treatment consisted of ampicillin with sulbactam (22 mg/kg, TID, IV). Three days after hospitalization, the patient developed hyperthermia and leukocytosis, and a marked edema in the left pelvic limb accompanied by hematoma formation was observed at the venous access site. The venous access was removed and sent to bacterial culture. Antimicrobial therapy was adjusted to clindamycin (11 mg/kg, TID, IV) and enrofloxacin (10 mg/kg, SID, IV) and escalated to include amikacin and ampicillin-sulbactam on the eleventh day. Due to the progressive clinical deterioration, the owner elected to proceed with euthanasia. Informed consent was obtained from the owner for this case report but not for a necropsy.

### 4.3. Case 3

A three-month-old mixed-breed female dog (BR250) was admitted to the emergency sector of the veterinary hospital with a history of vehicular trauma. The patient presented with open fractures in the hind limbs and underwent bilateral hind limb amputation due to extensive tissue devitalization and signs of contamination near the surgical sites. A Penrose drain was placed, and wound dressings were applied. For the first four days post-surgery, the dog was treated with amoxicillin with clavulanic acid (0.6 mL, BID, IV) and metronidazole 5 mg/mL (8.4 mL, BID, IV). On the fourth day of hospitalization, purulent secretion was observed at the site of the Penrose drain. The antimicrobial therapy was adjusted to ceftriaxone 1 g diluted in 10 mL (1.10 mL, BID, IV) and a swab of the secretion was surgically collected and submitted for bacterial culture. The dog remained hospitalized in the ICU for 18 days, receiving pain management, antimicrobial therapy, and local treatment of the surgical site, being released after a total of 35 days of hospitalization.

### 4.4. Bacterial Culture, Identification and Antimicrobial Resistance Tests

Samples collected from the three cases (swab of endotracheal tube secretion, swab from the venous access site on the left pelvic limb, and Penrose drain secretion swab from the left pelvic limb) were plated onto Brain Heart Infusion agar (BHI, Oxoid, Hampshire, UK) supplemented with equine blood and MacConkey agar (Kasvi, Pinhais, Brazil). Species identification was performed using MALDI-ToF as previously described [82] and antimicrobial susceptibility testing was performed using the disk diffusion method following the M100-Ed31 guidelines [83], with *Escherichia coli* ATCC^®^ 25922 used as the control. Isolates resistant to three or more antimicrobial classes were classified as multidrug-resistant [5].

For the isolate in Case 1 (B145), the following antimicrobials were tested: amoxicillin/clavulanic acid (30 μg), ampicillin (10 μg), cefoxitin (30 μg), ceftiofur (30 μg), cefotaxime (30 μg), cephalothin (30 μg), ciprofloxacin (5 μg), doxycycline (10 μg), enrofloxacin (5 μg), neomycin (30 μg), gentamicin (30 μg), nitrofurantoin (300 μg), trimethoprim-sulfamethoxazole (25 μg), ertapenem (10 μg), florfenicol (30 μg), and oxytetracycline (30 μg). Furthermore, an Etest (bioMérieux, Marcy l’Étoile, France) for ertapenem and colistin was performed in accordance with the manufacturer’s instructions. The MIC was determined as the lowest concentration showing complete inhibition of bacterial growth, as described by the Clinical and Laboratory Standards Institute (CLSI) [83].

For Cases 2 and 3 (B146 and BR250, respectively), seven antimicrobials were tested, each representing seven distinct classes: ciprofloxacin (5 μg), tetracycline (30 μg), gentamicin (10 μg), trimethoprim-sulfamethoxazole (25 μg), imipenem (10 μg), piperacillin-tazobactam (100/10 μg), and ceftazidime (30 μg). Similar to Case 1, an Etest (bioMérieux, Marcy l’Étoile, France) for meropenem and colistin was performed and interpreted according to the CLSI.

### 4.5. Whole Genome Sequencing and Comparative Genomics

The strains were incubated on Mueller–Hinton agar (Kasvi, Pinhais, Brazil) at 37 °C for 24 h. Genomic DNA was extracted using the Wizard Genomic DNA Purification Kit (Promega, EUA, Madison, WI, USA). Genome sequencing was performed using the Illumina MiSeq platform (mid-out 2 × 150 bp cycles). The raw data were analyzed using FastQC (Babraham Bioinformatics, Cambridge, UK), retaining only paired reads with a phred quality of 30 or higher.

The assembly was performed using SPAdes 3.5.0 in the careful mode [84,85]. GAP filling and polishing were performed using Pilon 1.24 [86]. The genomes have been deposited in the National Center for Biotechnology Information (NCBI) under BioProject accession number PRJNA1255613.

ResFinder 4.6 [87,88] was used to identify acquired antimicrobial resistance determinants and MobileElementFinder v1.0.3 [89] was used to identify mobile genetic elements. Kaptive 2.0 was used to type the capsule (K) and outer lipopolysaccharide (O) loci of *K. pneumoniae* [90]. Multilocus sequence typing (MLST) was performed using the Pasteur scheme, with sequence type (ST) determined through the PubMLST database [91].

The Singles nucleotide polymorphism (SNP) analysis was performed in CSIPhylogeny [92,93] with a minimal Z-score of 1.96 and a minimal depth at a SNP position of 10x. *A. baumannii* from ST15 (BioSample accession number SAMN14420901) and *K. pneumoniae* from ST340 (BioSample accession number SAMN05193406) were used as references [21,94]. Brazilian *A. baumannii* strains from human clinical isolates, including major carbapenemase-producing clones prevalent in Brazil (ST15 and ST79) as well as globally disseminated clones circulating in the country (ST1 and ST2), were obtained from the Bacterial and Viral Bioinformatics Resource Center (BV-BRC, available at https://www.bv-brc.org/) database [63,95] and used for comparative analysis with the isolates described in this study. Additionally, Brazilian human *K. pneumoniae* strains of ST340 (CC258) were obtained from the same database and included in the comparison with our *K. pneumoniae* isolate (Supplementary File S1). The phylogenetic tree was generated using iTOL v.6 online, applying midpoint rooting [28].

## 5. Conclusions

In this study, we report the occurrence of carbapenemase-producing *K. pneumoniae* and *A. baumannii* in three dogs hospitalized at a veterinary facility. This is the first description of *K. pneumoniae* ST340 and *A. baumannii* ST15 in animals. The detection of the major carbapenemase genes *bla*_KPC-2_ and *bla*_OXA-23_ highlights the potential role of companion animals in the dissemination of critical antimicrobial resistance determinants. Moreover, the close phylogenetic relationship between our isolates and clinical human strains underscores the need for One Health-focused studies to further investigate the epidemiology of globally relevant pathogens.

## Data Availability

Part of the data presented in this study are openly available in GenBank under BioProject accession number PRJNA1255613. The remaining data are provided in the three supplementary files accompanying this article.

## Supplementary Materials

The following supporting information can be downloaded at: https://www.mdpi.com/article/10.3390/antibiotics14060584/s1, Supplementary File S1; Supplementary File S2; Supplementary File S3.
