# Supplementary material for: Genomic Characterization of Carbapenem-Resistant Acinetobacter baumannii (OXA-23) and Klebsiella pneumoniae (KPC-2) Causing Hospital-Acquired Infections in Dogs
Source: Antibiotics (Basel). 2025 Jun 6;14(6):584. doi: 10.3390/antibiotics14060584 (PMC12189288; doi:10.3390/antibiotics14060584)
Supplement: Supplementary file 1 [file antibiotics-14-00584-s001.zip › Supplementary File S2.pdf]

Supplementary File S2–Mobile genetic elements in the sequenced genomes

**Isolate**	**Case**	**Bacterial species**	**Relevant AMRs in Plasmids**		**Chromosomally encoded virulence genes**	**Insertion sequences**	
			**Plasmid ID**	**AMR Genes**		**ISs**	**AMR Genes**
B145	1	*Klebsiella pneumoniae*	Col(pHAD28)	N/A	*fimH*, *mrkA*, *traT*, *iutA*, *clpK1*	ISCfr1	*aac(3)-IId*
			Col440I	*qnrB19*		ISKox1	N/A
			IncFIB(K)	N/A		IS102	N/A
			IncFII(K)	N/A		ISEcl1	N/A
			IncQ1	*aph(3')-VIa*, *blaKPC-2*		ISKpn1	N/A
						ISKpn18	N/A
			IncX10	N/A		ISKpn26	N/A
						ISKpn28	N/A
B146	2	*Acinetobacter baumannii*	N/A	N/A	N/A	ISAba14	N/A
						ISAba1	N/A
BR250	3	*Acinetobacter baumannii*	N/A	N/A	N/A	ISVsa3	*sul2*
						ISAba1	N/A
						ISAba125	N/A
						ISAba31	N/A
						ISOur1	N/A

Legend: AMR: antimicrobial resistance; N/A: not applicable
